# Liposarcoma: exploration of clinical prognostic factors for risk based stratification of therapy

**DOI:** 10.1186/1471-2407-9-205

**Published:** 2009-06-26

**Authors:** Hyo Song Kim, Jeeyun Lee, Seong Yoon Yi, Hyun Jung Jun, Yoon-La Choi, Geung Hwan Ahn, Sung Wook Seo, Do Hoon Lim, Yong Chan Ahn, Joon Oh Park, Sung Joo Kim

**Affiliations:** 1 Division of Hematology-Oncology, Department of Medicine, Samsung Medical Center, Sungkyunkwan University School of Medicine, Seoul, Korea; 2 Department of Pathology, Samsung Medical Center, Sungkyunkwan University School of Medicine, Seoul, Korea; 3 Department of Orthopedic Surgery, Samsung Medical Center, Sungkyunkwan University School of Medicine, Seoul, Korea; 4 Department of Radiation Oncology, Samsung Medical Center, Sungkyunkwan University School of Medicine, Seoul, Korea; 5 Department of Surgery, Samsung Medical Center, Sungkyunkwan University School of Medicine, Seoul, Korea; 6 Division of Medical Oncology, Yonsei Cancer Center, Yonsei University College of Medicine, Seoul, Korea

## Abstract

**Background:**

Prognosis and optimal treatment strategies of liposarcoma have not been fully defined. The purpose of this study is to define the distinctive clinical features of liposarcomas by assessing prognostic factors.

**Methods:**

Between January 1995 and May 2008, 94 liposarcoma patients who underwent surgical resection with curative intent were reviewed.

**Results:**

Fifty patients (53.2%) presented with well differentiated, 22 (23.4%) myxoid, 15 (16.0%) dedifferentiated, 5 (5.3%) round cell, and 2 (2.1%) pleomorphic histology. With the median 14 cm sized of tumor burden, about half of the cases were located in the retroperitoneum (46.8%). Seventy two (76.6%) patients remained alive with 78.1%, and 67.5% of the 5- and 10-year overall survival (OS) rates, respectively. Low grade liposarcoma (well differentiated and myxoid) had a significantly prolonged OS and disease free survival (DFS) with adjuvant radiotherapy when compared with those without adjuvant radiotherapy (5-year OS, 100% vs 66.3%, P = 0.03; 1-year DFS, 92.9% *vs *50.0%, respectively, P = 0.04). Independent prognostic factors for OS were histologic variant (P = 0.001; HR, 5.1; 95% CI, 2.0 – 12.9), and margin status (P = 0.005; HR, 4.1; 95% CI, 1.6–10.5). We identified three different risk groups: group 1 (n = 66), no adverse factors; group 2, one or two adverse factors (n = 28). The 5-year OS rate for group 1, and 2 were 91.9%, 45.5%, respectively.

**Conclusion:**

The histologic subtype, and margin status were independently associated with OS, and adjuvant radiotherapy seems to confer survival benefit in low grade tumors. Our prognostic model for primary liposarcoma demonstrated distinct three groups of patients with good prognostic discrimination.

## Background

Liposarcoma is the most common histology of soft tissue sarcoma and accounts for 20% to 30% of all sarcoma in adults [1-3]. Despite the skeletal and soft tissue are the most abundant tissue in the human body, soft tissue sarcoma consists of only 1% of neoplasms. Due to this low incidence of the soft tissue sarcoma, only a few studies reported some prognostic factors for liposarcoma, and optimal treatment strategies and thereby prognoses have not been fully defined yet [4-8].

Clinical behavior and prognostic features of soft tissue sarcoma are known to be associated with histology, and anatomic distribution. Conventionally, low grade lesions have higher incidence of local recurrence, and high grade tumors present with higher incidence of local recurrence and distant metastasis. Although surgical resection also remains as the primary treatment of all soft tissue sarcoma [7,9], radiotherapy has been extensively used in the treatment of sarcomas to improve cure rate and patients' quality of life [10]. However, there are no confirmatory studies which directly compared preoperative with postoperative radiotherapy. Among them, ongoing Toronto study group's trial demonstrated preoperative radiotherapy had slightly better outcome in terms of morbidity and survival benefit even the study was underpowered [11]. There is still much controversy about the indications and appropriate dose/timing for perioperative radiotherapy. Patients with high risk features for recurrence should be offered of pre- or postoperative treatment to further improve treatment outcome.

In this study, we analyzed clinicopathologic features and prognostic factors for the histologically confirmed liposarcoma patients. We further attempted to devise a prognostic model specific for the disease in order to facilitate decision making in liposarcoma.

## Methods

### Patients

Between January 1995 and May 2008, a total of 105 patients were diagnosed as liposarcoma by histologic exams at Samsung Medical Center. This retrospective study was reviewed and approved by the Samsung Medical Center institutional review board (Seoul, Korea). Among them, 94 patients were treated by surgical resection with curative intent. The other 11 patients were not available for further analyses along with complete clinical data due to patients' refusal for surgical resections or referrals after the relapses. Descriptive characteristics for all the 94 patients are listed in Table 1. Liposarcoma was classified into 5 histologic subgroups based on Evans Classification [12]; well-differentiated, myxoid, dedifferentiated, round, and pleomorphic. No patient had distant metastasis at the time of surgical resection.

**Table 1:** Patient characteristics

Variables	No. of patients		
	Total (n = 94) (%)	Low (n = 72)	High (n = 22)
**Age, Median (range)**	**56 (20–80)**	55 (20–80)	58.5 (24–75)
**Gender**			
Male	**53 (56.4%)**	42 (58.3%)	11(50.0%)
Female	**41 (43.6%)**	30 (41.7%)	11(50.0%)
**Presentation status**			
Biopsy	**49 (52.1%)**	38 (52.8%)	11(50.0%)
No prior biopsy or treatment	**20 (21.3%)**	17 (23.6%)	3(13.6%)
Prior excision	**25 (26.6%)**	17 (23.6%)	8(36.4%)
**Histologic variant**			
Well differentiated	**50 (53.2%)**	-	-
Myxoid	**22 (23.4%)**	-	-
Dedifferentiated	**15 (16.0%)**	-	-
Round	**5 (5.3%)**	-	-
Pleomorphic	**2 (2.1%)**	-	-
**Primary site**			
Retroperitoneum	**44 (46.8%)**	31(43.0%)	13 (59.1%)
Without contiguous organ resection	**32 (34.0%)**	23 (31.9%)	9 (40.9%)
With contiguous organ resection	**12 (12.8%)**	8 (11.1%)	4 (18.2%)
Lower extremity	**24 (25.5%)**	23 (31.9%)	1(4.5%)
Upper extremity	**9 (9.6%)**	9 (12.5%)	0 (0)
Trunk	**17 (18.1%)**	9 (12.5%)	8 (36.4%)
**Tumor burden**			
Median (cm, range)	**14 (2–48)**	13 (2–45)	15 (2–48)
**Margins**			
Negative margins	**50 (53.2%)**	42 (58.3%)	8 (36.4%)
Positive micro margins	**32 (34.0%)**	24 (33.3%)	8 (36.4%)
Positive gross margins	**12 (12.8%)**	6 (8.3%)	6 (27.3%)
**Postoperative radiotherapy for primary disease**			
Median dose (cGy)	**5300** **(2500–6000)**	5400 (2500–6000)	5000 (2600–6000)
Yes	**31 (33.0%)**	22 (30.5%)	9 (40.9%)
Negative/microscopic/gross positive (%)	**17/10/4** **(54.8/32.3/12.9)**	13/8/1 (59.1/36.4/4.5)	4/2/3 (44.4/22.2/33.3)
No	**63 (67.0%)**	50 (69.4%)	13 (59.1%)

Primary sites were categorized into lower extremity, upper extremity, trunk, retroperitoneum with or without contiguous organ resection. A primary site was considered as upper extremity when it was at or beyond the shoulder joint, and as lower extremity when it was located in the groin, thigh, or leg. Retroperitoneal tumors were categorized as with or without contiguous organ resection (whether any of the following resections were performed: colon, small bowel, pancreas, spleen, bladder, uterus) according to the surgical record. Tumor burden was determined by the maximum diameter of the primary tumor at the time of surgical resection. The type of surgery was reviewed from the surgeons' assessment as documented on the operative report. We analyzed specimen margins by gross and microscopic examinations in terms of six dimensions (superior, inferior, medial, lateral, anterior, and posterior). We defined a clear margin when there was no tumor at least 1 mm or more from the edge of the inked specimen. In addition, microscopically positive margin was defined when there was a tumor within ≤ 1 mm of the edge. Specimen margins were categorized as clear, microscopically positive, or grossly positive. None of the patient had received adjuvant chemotherapy. Thirty-one (33.0%) patients underwent postoperative external beam radiation therapy. Follow-up data included recurrence (local or distant metastasis), and vital status (alive without disease, alive with diseases, dead of other cause, dead as a result of sarcoma or sarcoma treatment).

### Statistics

The primary end point was overall survival (OS), which was calculated using Kaplan-Meier method. OS was defined from the date of surgery to date of death related to the disease or complication. Survival rates were compared for statistical differences using log-rank test. OS was assessed with respect to following factors: age (both as a continuous variable and divided at the median), sex, histologic subtype, presentation status, primary site, tumor burden (both as a continous variable and divided into two groups), and resection margin (negative, microscopically positive, grossly positive). We also analyzed 5- and 10-year OS. Multivariate analysis was performed using stepwise Cox proportional hazards regression modeling. P values less than 0.05 were considered statistically significant and all P values corresponded to two-sided significance tests. The disease free survival was defined as time from date of surgery to date of first recurrence for patients with negative or microscopically positive resection margin.

## Results

### Patient characteristics

Ninety-four liposarcoma patients who underwent surgical resection with curative intent were included in the analysis (Tab 1). There were 41 females and 53 males with median age of 56 years. The median tumor burden was 14 cm (range, 2–48). The distribution of histologic subtypes were as follows: well differentiated in 50 patients (53.2%), myxoid in 22 patients (23.4%), dedifferentiated 15 patients (16.0%), round cell 5 patients (5.3%), and pleomorphic in 2 patients (2.1%). We classified the histologic grade into two categories according to previous study [13]. There were 72 patients with low grade (well-differentiated and myxoid tumors) and 22 patients with high grade tumors (dedifferentiated, round cell, pleomorphic tumors). Fifty patients had negative resection margins, 32 had microscopically positive margins, and 12 had grossly positive margins. Nearly half of the tumors were located in the retroperitoneum; one third of these patients required resection of contiguous organ other than kidney. We also analyzed clinical characteristics according to the histologic grade (low *vs *high) as shown in Table 1.

### Survival analyses

**Figure 1:**
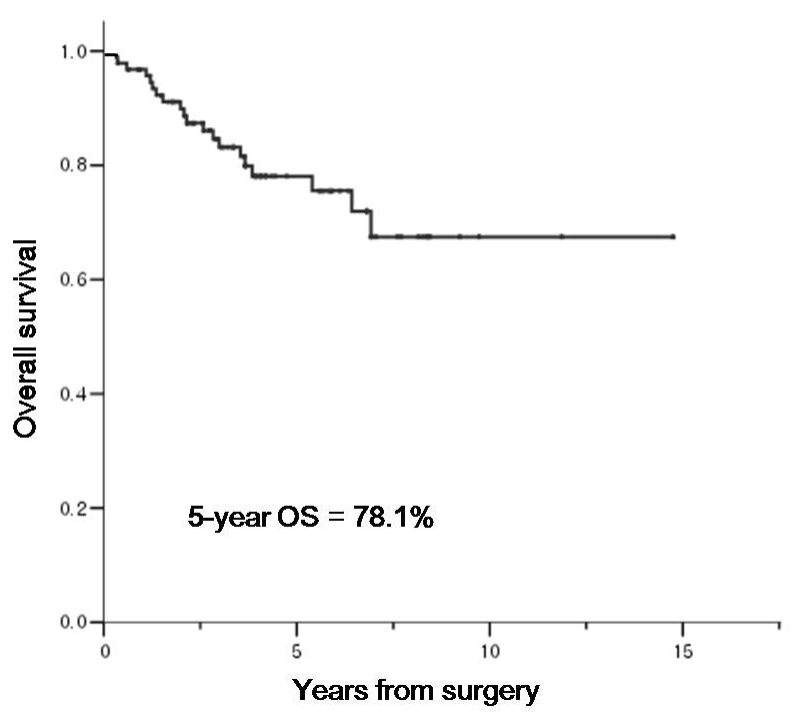
**Overall survival of all patients**.

After a median follow-up duration of 48 months (range, 4.5–166.4 months), 72 (76.6%) patients remained alive and 22 (23.4%) patients died of the disease. The 5- and 10-year overall survival probabilities were 78.1% and 67.5%, respectively (Figure 1).

OS according to histologic subtype is shown in Table 2. OS was significantly different according to the histologic subtype (P < 0.001). Histologic subtypes were dichotomized into two groups (well-differentiated and myxoid tumors *vs *others). The 5-year OS rates for low and high grade tumors were 86.1%, and 48.6%, respectively. While patients with microscopically negative and positive margins had 5-year OS of 88.1% and 75.0%, respectively, patients with grossly positive margins had a significantly decreased 5-year OS of 44.0%. For the tumor burden, patients with tumor diameter less than 10 cm had significantly prolonged OS compared to those with greater than 10 cm (P = 0.013). The 5-year OS rates were 100% and 67.3% for patients with tumor diameter less and greater than 10 cm, respectively. For the primary sites, extremity site showed the most favorable prognosis (upper extremity, 5-year OS, 100%; lower extremity, 86.5%) while retroperitoneal tumors demonstrated a reduced 5-year OS rate of 61.1%. There was no significant difference in terms of median age (P = 0.25), sex (P = 0.64), and between those with or without contiguous organ resection (P = 0.19).

**Table 2:** Analysis of Histologic Subtypes in 5-Year Overall Survival (OS)

Histologic Variant	Total	No. of death	5-Year OS (%)	*P*
**Well differentiated**	50	5 (10%)	93.3	P < 0.001
**Myxoid**	22	4 (18.2%)	75.7	
**Dedifferentiated**	15	6 (40%)	54.5	
**Round**	5	3 (60%)	40.0	
**Pleomorphic**	2	2 (100%)	0	

For histologic status, 20 (90.9%) and 2 (9.1%) patient of low grade liposarcoma experienced local recurrence and distant metastasis, compare to 11 (84.6%) for local recurrence and 2 (15.4%) for distant metastasis among high grade patients. In terms of resection margins, 23 patients out of 27 patients (85.2%) with negative resection margin developed local recurrence, meanwhile all the 8 patients with positive resection margin experienced local recurrence.

We also evaluated DFS only in 82 patients with negative and microscopically positive margins. Among the evaluable 71 patients, 27 patients (38.0%) experienced relapse, in which 4 (14.8%) distant metastasis and 23 (85.2%) local recurrences. Thirteen patients had received excision of recurred lesion for curative intent. The median DFS was 17.6 months (95% confidence interval [CI], 14.5–20.6 months) for all patients. The DFS was significantly different according to the histologic subtype (P = 0.049). The median DFS of low and high grade tumors were 42.4 and 26.8 months, respectively. The 1-year DFS rates for low and high grade tumors were 83.3%, and 44.4%, respectively. There were not significantly different DFS in terms of resection margin (P = 0.23), primary site (P = 0.25), and tumor burden (P = 0.80).

### Impact of adjuvant radiotherapy on survival

**Figure 2:**
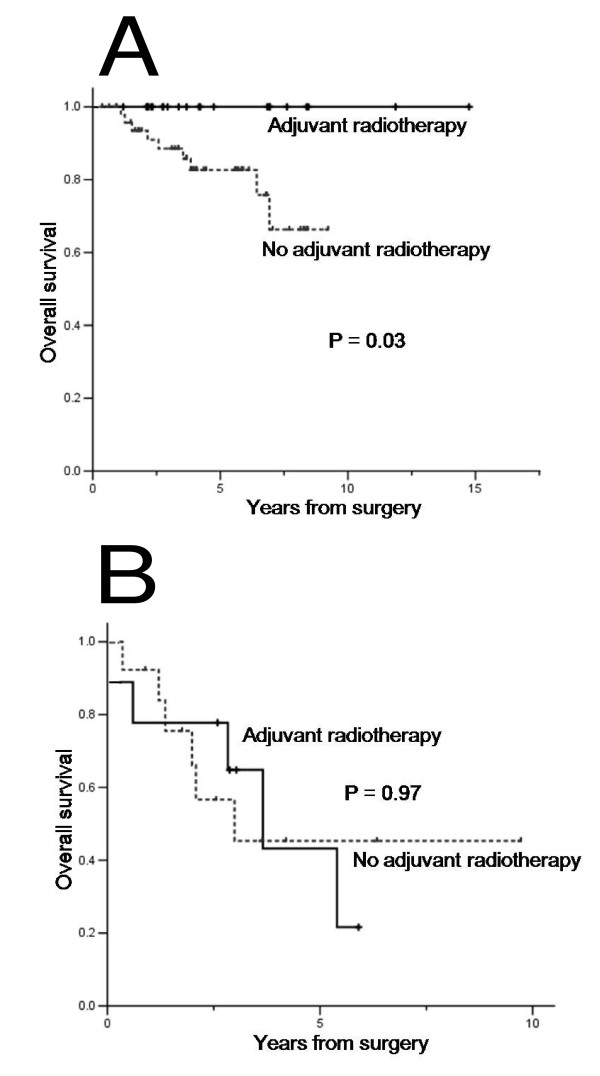
**Overall survival according to adjuvant radiotherapy for patients with low grade sarcomas (A) and high grade liposarcomas (B)**.

In all patients, the administration of adjuvant radiotherapy did not seem to confer survival benefit (P = 0.39). We further performed subgroup analyses to identify patients who may benefit most from postoperative adjuvant radiotherapy. In low grade liposarcoma (well differentiated, and myxoid), a subgroup with adjuvant radiotherapy (n = 22) demonstrated significantly prolonged OS when compared with those without adjuvant radiotherapy (n = 50) (5-year OS, 100% *vs *82.7%, respectively, P = 0.03, Figure 2A). In contrast, there was no survival difference between the adjuvant radiotherapy (+) and (-) group with high grade liposarcoma (5-year OS, 45.3% *vs *43.2%, P = 0.97, Figure 2B). Of note, there was a trend toward worse local control rate (22.7% *vs *55.6%, P = 0.08) following adjuvant radiotherapy in high grade tumors when compared with low grade liposarcomas. However, there was no significant difference respect to adjuvant radiotherapy (+) and (-) group among in patients with negative resection margins (n = 50, P = 0.42) and micro/macroscopically positive resection margin (n = 44, P = 0.61).

For DFS, in low grade liposarcoma, patients received adjuvant radiotherapy (n = 21) demonstrated significantly prolonged DFS when compared with those without adjuvant radiotherapy (n = 46) (1-year DFS, 92.9% *vs *50.0%, respectively, P = 0.04). Similary, there was no survival difference between the adjuvant radiotherapy (+) and (-) group with high grade liposarcoma (P = 0.67).

### Prognostic model for patients treated by surgical resection with curative intent

**Figure 3:**
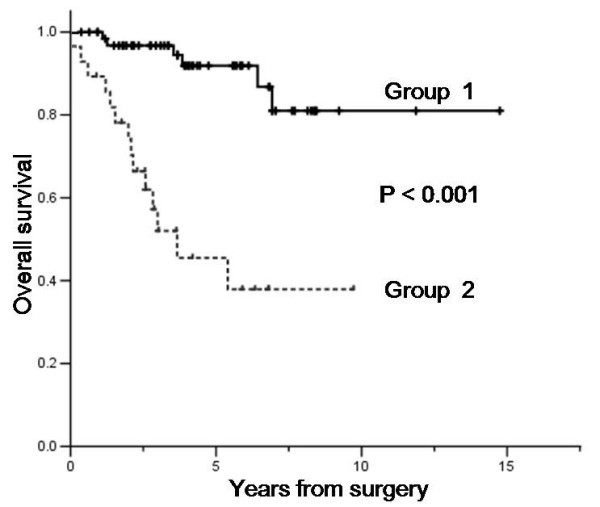
**Overall survival of patients by prognostic model**. Group 1 (n = 66), no adverse factors; Group 2, one or two adverse factors (n = 28).

The clinical factors predicting poor survival at univariate analyses were as follows: histologic variant (high grade, P < 0.001), status of resection margin (microscopically or grossly positive margin, P < 0.001), primary site (retroperitoneum or deep trunk, P = 0.009) and tumor burden (more than 10 cm, P = 0.013). The forward Cox regression model was used to establish independent prognostic factors. Independent prognostic factors for survival were histologic variant (P = 0.001; HR, 5.1; 95% CI, 2.0 – 12.9), and margin status (P = 0.005; HR, 4.1; 95% CI, 1.6–10.5) as shown in Table 3. All the patients had complete information on the two parameters and included in the prognostication. The prognostic grouping was performed according to the following criteria: group 1 (n = 66), no adverse factors; group 2, one or two adverse factors (n = 28). The Kaplan-Meier survival curves according to the prognostic index are shown in Figure 3 (P < 0.001). The prognostic model separated patients into two risk groups with significantly different survival outcomes. The 5-year OS rate for group 1, and 2 were 91.9%, 45.5%, respectively.

**Table 3:** Prognostic factors for survival in Multivariate Analysis

Variables	Univariate	Multivariate		Survival (%)
		
	*P*	HR (95% CI)	*P*	5-year OS
**Histologic variant **(high *vs *Low)	< 0.001	5.1 (2.0–12.9)	0.001	48.6 *vs *86.1
**Margin status **(positive *vs *negative)	< 0.001	4.1 (1.6–10.5)	0.005	44.0 *vs *83.0
**Primary site** (trunk or retroperitoneum *vs *extremity)	0.009	-	0.239	61.1 *vs *90.0
**Tumor burden **(> 10 cm *vs *≤ 10 cm)	0.013	-	0.164	67.3 *vs *100.0

## Discussion

Liposarcoma is the most common soft tissue sarcoma which accounts for about 20% of sarcoma in adults [14]. Histologic classification have been well established by the current World Health Organization, which is almost identical to the paradigm implemented by Enzinger and Winslow [15]. The 5 histologic subgroups of liposarcoma pursue different natural clinical course not only in terms of clinical features but also the survival outcome. Nevertheless, current clinical practice is not optimized according to different histologic subtypes and clinical protocol often does not reflect such difference. In this study, we retrospectively analyzed a homogeneous cohort of primary liposarcoma patients. To improve the risk based stratification for treatment, we also attempted to establish a prognostic model for this subset of patients.

In the present study, the most common subtype was well-differentiated liposarcoma which accounted for approximately 50% of all cases. The overall survival was well stratified by histologic subtype, which was concordant with the results from previous studies [13,16]. Multivariate analyses further demonstrated that histologic subtype was an independent prognostic factor for survival. High grade tumors (dedifferentiated, round cell, pleomorphic tumors) were associated with approximately 5-fold increased risk of deaths compared with low grade tumors.

The efficacy of radiation therapy to improve local control has been well reported in the previous randomized trials of extremity soft tissue sarcomas [17,18]. In contrast to extremity sarcoma, the role of adjuvant radiotherapy for patients with retroperitoneal sarcoma is not well clarified. Some studies revealed that adjuvant radiotherapy reduces the risk of local recurrence and improves local control [19,20]. Thereby some patient with negative resection margin had received adjuvant radiotherapy when primary tumors are large-sized or deep retroperitoneal origin. The addition of adjuvant radiotherapy seems to confer survival benefit in low grade tumors although limited by retrospective nature of the analyses. As illustrated in Figure 2A, the impact of adjuvant radiotherapy on survival was more prominent in low grade sarcomas than high grade tumors. Interestingly, the local control rate was considerably lower in the high grade tumor group when compared with low grade group following postoperative radiotherapy (local failure rate 22.7% *vs *55.6%, P = 0.08). In support of this, there were no differences in margin status between the two groups. In addition, to clarify the benefit of radiotherapy, we also analyzed significant prognostic factors for DFS and efficacy of adjuvant radiotherapy for patients with negative and microscopically positive margins. The DFS was significantly different according to the histologic subtype, and patients with low grade liposarcoma demonstrated better DFS with postoperative adjuvant radiotherapy. The underlying mechanisms that cause these changes are uncertain. It is possible that patients with high grade tumor had slightly more retroperitoneal site and positive resection margins than those with low grade. Even though this is the retrospective study with relatively small sample size, we emphasize that these results should be cautiously interpreted and prompt future prospective studies.

To improve risk-based stratification for therapy, we attempted to establish a prognostic model specifically devised for patients treated by surgical resection with curative intent. At multivariate analyses, histologic variant (P = 0.001; HR, 5.1; 95% CI, 2.0–12.9), resection margin status (P = 0.005; HR, 4.1; 95% CI, 1.6–10.5) retained statistical significance. The prognostic grouping was based on scoring system of the adverse factors and yielded distinctive sets of two groups with different survival outcome (5-year OS rates for group 1, and 2 were 91.9%, and 45.5%, respectively.) Given a poor prognosis of patients with one or two adverse factors in the model, this subgroup of patients may potentially benefit from more aggressive postoperative treatment. Hence, the role of adjuvant chemotherapy with or without radiotherapy may be studied in this subgroup of patients. Our prognostic model demonstrated two groups of patients with distinct prognostic discrimination. More aggressive postoperative treatment such as combined modalities with chemotherapy and radiotherapy should be offered in the context of clinical trials in this particular subgroup of liposarcoma patients. Nonetheless, prospective study is needed to validate the model on larger population.

In this study, the frequency of retroperitoneal primary site was high accounting for half of the cohort. Previous studies in western population demonstrated relatively lower incidence of retroperitoneal primary site ranging from 25–33.4% of all cases [4,13]. Retroperitoneal tumors usually presented with large-sized mass and involvement of multiple contiguous organs and thus, a wide excision with adequate resection margin may not have been technically feasible. Its anatomic characteristics may render relatively poor survival outcome of this study compare to previous studies which have shown 75% to 83% of 5-year overall survival.

## Conclusion

Histologic subtype, and margin status are independently associated with disease specific survival, and patients with low grade histology had benefit from adjuvant radiotherapy. Proposed model for primary liposarcoma demonstrated distinct groups of patients with good prognostic discrimination. The impact of adjuvant radiotherapy on OS and DFS were more prominent in low grade sarcomas than high grade tumors. We hope our study may facilitate further prospective study and alternatively clinical decision making in liposarcoma, combined with reliable molecular markers.

## Competing interests

The authors declare that they have no competing interests.

## Authors' contributions

HSK drafted the manuscript. HSK, SYY, HJJ, YLC, GHA, SWS, DHL, YCA, SK collected the data and performed the statistical analysis. SWS, DHL, YCA, SK, JL and JOP followed the patients. SK, JOP, and JL designed the study and helped with the manuscript. All authors read and approved the final manuscript.

## Pre-publication history

The pre-publication history for this paper can be accessed here:

http://www.biomedcentral.com/1471-2407/9/205/prepub
